# ABC transporter research: going strong 40 years on

**DOI:** 10.1042/BST20150139

**Published:** 2015-10-09

**Authors:** Frederica L. Theodoulou, Ian D. Kerr

**Affiliations:** * Biological Chemistry and Crop Protection Department, Rothamsted Research, Harpenden, AL5 2JQ, U.K.; † School of Life Sciences, Queen's Medical Centre, University of Nottingham, Nottingham, NG7 2UH, U.K.

**Keywords:** ATP-binding cassette (ABC) transporter, disease, multi-drug resistance, protein structure, transport mechanism

## Abstract

In most organisms, ABC transporters constitute one of the largest families of membrane proteins. In humans, their functions are diverse and underpin numerous key physiological processes, as well as being causative factors in a number of clinically relevant pathologies. Advances in our understanding of these diseases have come about through combinations of genetic and protein biochemical investigations of these transporters and the power of *in vitro* and *in vivo* investigations is helping to develop genotype–phenotype understanding. However, the importance of ABC transporter research goes far beyond human biology; microbial ABC transporters are of great interest in terms of understanding virulence and drug resistance and industrial biotechnology researchers are exploring the potential of prokaryotic ABC exporters to increase the capacity of synthetic biology systems. Plant ABC transporters play important roles in transport of hormones, xenobiotics, metals and secondary metabolites, pathogen responses and numerous aspects of development, all of which are important in the global food security area. For 3 days in Chester, this Biochemical Society Focused Meeting brought together researchers with diverse experimental approaches and with different fundamental questions, all of which are linked by the commonality of ABC transporters.

## A brief history of ABC transporters

**Figure 1:**
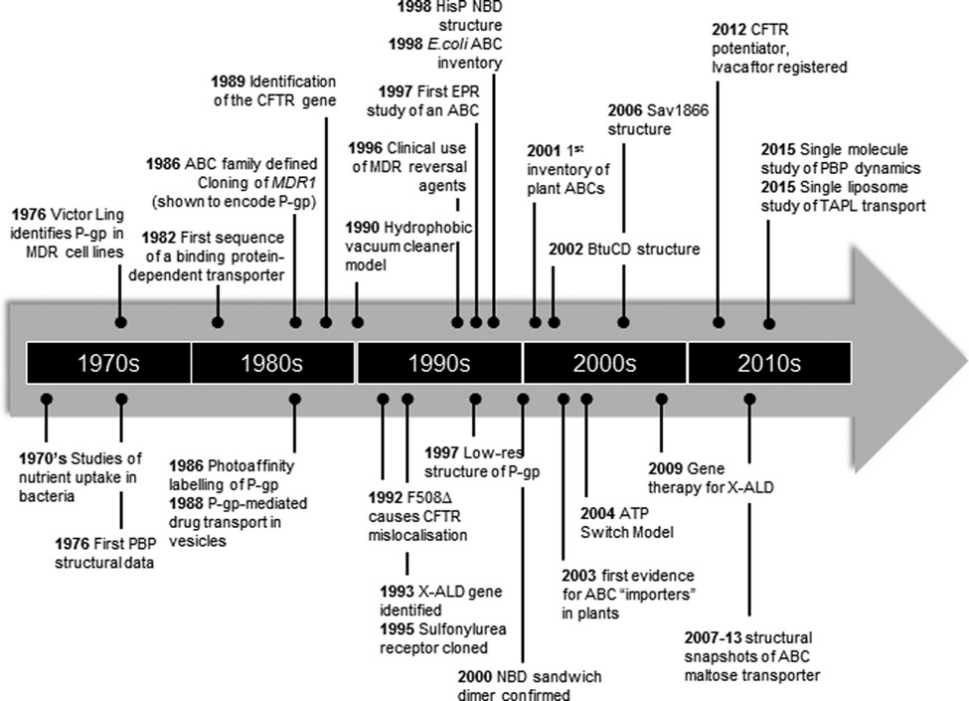
Timeline showing selected key discoveries and events in ABC research

The ATP-binding cassette (ABC) transporter field emerged from studies on nutrient uptake in bacteria in the 1970’s with the biochemical characterization of substrate-binding protein (SBP)-dependent transport systems energized directly by hydrolysis of ATP [1]. The early 1980’s saw the cloning of several genes encoding such transporters, spearheaded by the histidine permease of *Salmonella typhimurium* and maltose permease of *Escherichia coli* [2,3]. In parallel, medical researchers were tracking down the gene encoding permeability-glycoprotein/P-glycoprotein (P-gp), a large glycosylated membrane protein associated with multi-drug resistance in mammalian cells [4]. P-gp was eventually cloned in 1985 [5] and with increasing availability of cDNA sequences, it became apparent that both the mammalian and the bacterial transporters contained highly conserved nt-binding motifs (the Walker A and B sequences) [6], hinting at a common evolutionary origin. In 1986, it was recognized that these ATP-binding subunits defined a large superfamily of transport proteins [7–10] although the term ABC transporter was not actually coined until 1990 [11], by which time it was became apparent that ABC transporters were not only ubiquitous but also involved in diverse biochemical and physiological processes. A timeline of key events in the development of ABC transporter research is given in Figure 1 and a vivid account of the trials and tribulations of characterizing one of the prototypical ABC transporters (P-gp) is presented by Callaghan [12].

**Figure 2:**
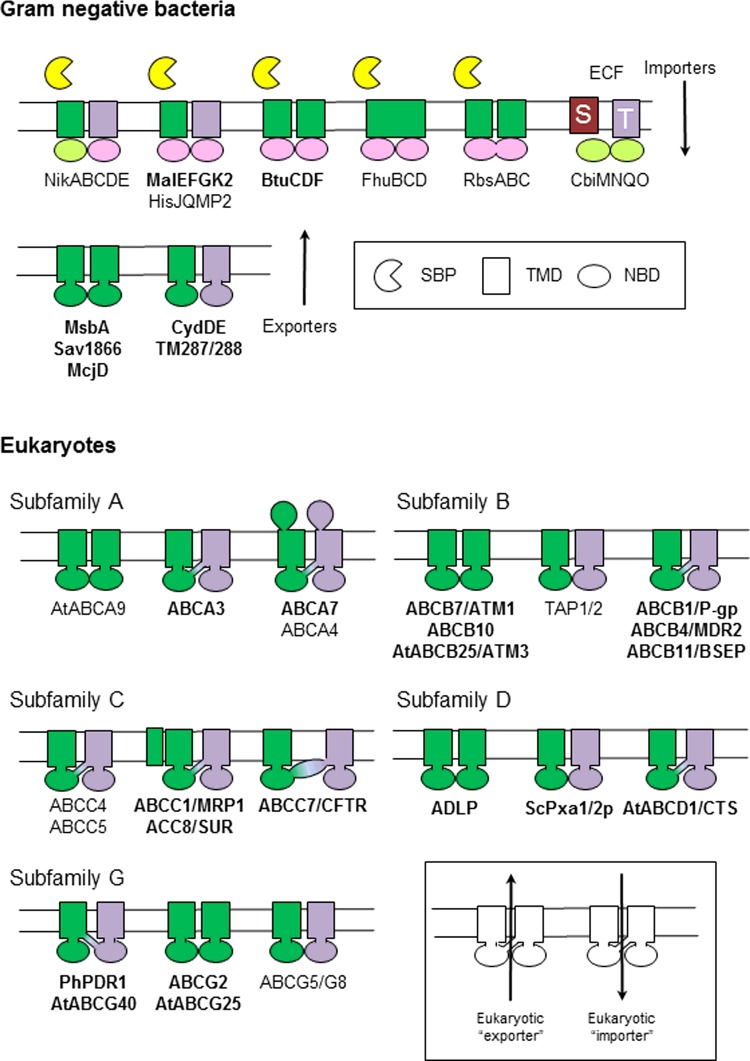
Domain organization of ABC transporters from different organisms In Gram-negative bacteria, importers comprise two TMDs, two NBDs (ATPase subunits) and a periplasmic SBP. In Gram-positive bacteria and archaea which have no outer membrane, SBPs are anchored to the extracellular face of the cytoplasmic membrane, or to the TMDs (not shown). Energy coupling factor (ECF) transporters comprise two ATPase subunits, a transmembrane protein (T) and a substrate-specific integral membrane protein (S). All bacteria and archaea also contain homo- or heterodimeric exporters, in which a TMD is fused with an NBD. Eukaryotic ABC proteins are classified in eight subfamilies (A–H). The organization of core and additional domains is shown for different representative topologies in each subfamily; transporters discussed in this volume are indicated in bold. Not included are soluble subfamilies E and F and transporter subfamily H, which is absent from mammals, plants and fungi. Plant genomes also encode several ‘prokaryotic-like’ ABC transporters, designated subfamily I, which have been acquired via the endosymbiotic origin of mitochondria and plastids [15] (not shown).

Genome sequencing, complemented by careful studies of individual transporters, has revealed a common architecture with a core ABC transporter comprising two nt-binding domains (NBDs) and two transmembrane domains (TMDs), often with additional domains. These domains are encoded either as individual proteins (typically in prokaryotes) or in varying degrees of fusion, with eukaryotic ABC proteins largely expressed as so-called ‘half-size’ (TMD–NBD or NBD–TMD) or ‘full-size’ (TMD–NBD–TMD–NBD or NBD–TMD–NBD–TMD) transporters (Figure 2) [13]. Domain organization and phylogenetic analysis have defined eight major subfamilies of eukaryotic ABC proteins, two of which (E and F) are not membrane associated and have functions other than transport [14,15]. Intriguingly, the ABC family includes both ‘exporters’ and ‘importers’ [16] (Figure 2). Importers, which typically rely on periplasmic SBPs to deliver substrates to the transporter core, were considered to be restricted to prokaryotes (and, rarely, organelles of endosymbiotic origin) but exporters are common to all kingdoms [15–17]. Previously, the discovery of a group of prokaryotic micronutrient uptake transporters dependent on ABC transporter NBDs but without extracytoplasmic SBPs (the energy coupling factor ABC transporters) has further extended the repertoire of ABC transporter architecture [18].

## Diversity of function: linking physiology with biochemistry

The functions of ABC proteins are too numerous to catalogue in detail here, but it is well established that they transport a huge range of diverse substrates, from simple ions, through polar, amphipathic and hydrophobic organic molecules to peptides, complex lipids and even small proteins. Consequently, ABC transporters are implicated in a wide array of developmental processes [14,15,17,19]. Plants have the largest complement of ABC proteins of all eukaryotes [15,19] and it was fitting that the meeting opened with a session devoted to diversity of function headlined by Enrico Martinoia [20], who described the roles of plant ABC transporters in phytohormone transport. The ABC superfamily expanded dramatically with the conquest of land by plants, associated in part with their sessile nature, multi-cellularity and extensive capacity for synthesis of secondary chemicals. It has long been hypothesized that plant ABC transporters would be involved in the synthesis and deposition of secondary metabolites (from which many important drugs are derived), but an important discovery has been the implication of subfamily G transporters in the transport of several major classes of plant hormones, namely: auxins, cytokinins, abscisic acid and the recently-discovered strigolactones [20]. Intriguingly, Martinoia and colleagues [19,20] drew attention to the increasing number of reports in the literature suggesting that both plant ABCGs and ABCBs can act as importers, challenging the ‘exporters only’ adage for eukaryotic ABC transporters (Figure 2). Much of this evidence is phenomenological and requires confirmation *in vitro* but perhaps the most convincing example is *Arabidopsis* ABCB14, which mediates malate uptake when expressed in *E.coli* and HeLa cells [21]. The ability of plant (and yeast) ABC research to make us rethink paradigms was also highlighted by Alison Baker and colleagues [22,23], who presented a novel mechanism for peroxisomal fatty acyl-CoA import by ABCD proteins, involving substrate cleavage catalysed by a thioesterase activity intrinsic to the transporter, followed by re-esterification by acyl activating enzymes which form a complex with the transporter in the peroxisomal lumen.

Much information about the roles of ABC transporters has been deduced using loss of function mutants isolated in genetic screens [20] and the pressing need for complementary biochemical studies of plant ABC transporters was outlined by François Lefèvre, using ABC sub-class G as an exemplar [24]. Some plant ABC transporters have been identified in multiple genetic screens and have been ascribed several different biochemical functions, based on their apparently disparate physiological roles. How do you go about characterizing a transporter when the identity of the transport substrate (or substrates) is not immediately obvious? An interesting approach to this question was provided by Janneke Balk and colleagues [25], who used a LC–MS metabolomic approach [26] to identify glutathione polysulfides as likely physiological substrates for *Arabidopsis* ABCB25/ABC transporter of mitochondria 3 (ATM3), a mitochondrial ABC transporter involved in iron-sulfur cluster and molybdenum cofactor assembly. Importantly, the physiological relevance of the transport studies was verified by follow-up genetic experiments [25,27]. Also exploring the functions of ABC transporters in redox processes, Mark Shepherd [28] proposed a role for the CydDC complex of *E. coli* in redox sensing and NO tolerance. CydDC exports cysteine and glutathione to the periplasm but is also thought to bind haem on the periplasmic surface which stimulates reductant export and may interact with gaseous signalling molecules, suggesting a novel virulence mechanism for bacteria.

## ABC transporters in health and disease: from bench to bedside and back again

As in plants and microbes, the importance of human ABC proteins and the diversity of their physiological roles are underscored by the consequences of their dysfunction. To date, over 20 ABC proteins, representing all sub-families have been associated with human disease (Table 1) and several others play clinically important roles in drug metabolism and resistance.

**Table 1:** ABC transporters associated with human health and disease References cited are from relevant papers in this Special Issue.

ABC transporter	Disease	References
ABCA1	Tangier disease and familial high density lipoprotein (HDL) deficiency; atherosclerosis; Alzheimer's disease	[38]
ABCA3	Neonatal surfactant deficiency and pulmonary fibrosis; congenital cataract	[37]
ABCA4	Stargardt macular degeneration	
ABCA7	Alzheimer's disease	[38]
ABCA12	Harlequin and lamellar ichthyosis	
ABCB1/transporter associated with antigen processing (Tap)2; ABCB2/Tap1	Immune deficiency; arthritis risk	
ABCB4/MDR2	PFIC3; other types of cholestasis	[39]
ABCB7	Sideroblastic amaemia and ataxia	[26]
ABCB11/bile salt export pump (BSEP)	PFIC2; intrahepatic cholestasis of pregnancy; neonatal respiratory distress syndrome	[39]
ABCC2/MRP2	Dubin–Johnson syndrome	
ABCC5/MRP5	Inherited hypertrichosis	
ABCC6/MRP6	Pseudoxanthoma elasticum	
ABCC7/CFTR	CF	[30,31]
ABCC8/SUR1	Diabetes	[35]
ABCC9/SUR2	Diabetes	[35]
ABCD1/adrenoleukodystrophy protein (ALDP)	X-linked adrenoleukodystrophy	[23,45]
	X-linked adrenomyeloneuropathy	
ABCD3/peroxisome membrane protein (PMP70)	Hepatosplenomegaly; liver disease	[23,45]
ABCD4/PMP69	Inborn error of vitamin B12 metabolism	[23]
ABCG2/breast cancer resistance protein (BCRP)	Gout and hyperuricaemia	
ABCG5; ABCG8	Sitosterolemia; coronary heart disease; gallstone disease	
ABCB1/P-gp; ABCC1/MRP1, ABCG2/BCRP	Multi-drug resistance	[12,44,45]
ABCC2-6	Drug transport	

It is now over 25 years since the causative gene in cystic fibrosis (CF) was identified as ABCC7 [also known as the CF transmembrane conductance regulator (CFTR)] [29] and a huge wealth of information has been obtained for this atypical ABC protein which is not in fact a primary active transporter but an ATP-gated chloride channel. David Sheppard emphasized the importance of understanding how different mutations affect CFTR function for the development of chemical correctors and discusses together with Pollock et al. [30,31] in this volume how species differences can be exploited to understand CFTR structure and function. To date, over 2000 mutations have been associated with CF [30], some of which affect key phosphorylation sites of CFTR [32]. Bibek Aryal [33] presented novel examples of ABC transporter regulation by phosphorylation and in this special issue, draws intriguing parallels between phosphorylation of the CFTR regulatory domain and phosphorylation of the linker region in plant ABCB transporters. The sulfonylurea receptors (SUR1/ABCC8; SUR2/ABCC9) are also atypical ABC proteins; by forming a hetero-octameric complex with potassium channel Kir6.2 subunits they produce the ATP-sensitive potassium channel whose activity is critical for insulin secretion [34,35]. Heidi de Wet [35] explained the complex mechanism of channel inhibition by sulfonylureas using elegant electrophysiological approaches and examined implications for treatment of neonatal diabetes.

A recurring theme of the meeting was the need for combinations of techniques to understand ABC transporter function/mis-function and role in physiology/disease, particularly for rare disorders and diseases with complex aetiologies. The involvement of ABCG2 in pre-disposition to gout is familiar to many in the ABC transporter community [36], but here we heard about studies implicating two members of the ABCA subfamily in disease. Andrea Masotti [37] outlined the challenges in diagnosing a spectrum of rare disorders of the surfactant system linked to mutations in the gene encoding the lung specific phospholipid transporter ABCA3 and Brett Garner [38] described how ABCA7 was identified as a genetic risk factor in late-onset Alzheimer's disease, presenting recent work with mouse and cell-based disease models to understand the function of ABCA7 in this context. The multi-disciplinary theme was continued by John Schuetz and Kenny Linton [39] who described work characterizing ABC transporters in liver disease. Owing to a combination of biochemistry, physiology, genetics and clinical science, the roles of three transporters: lipid floppase, ABCB4, bile salt export pump, ABCB11 and the P-type ATPase, ATP8B1 in progressive familial intrahepatic cholestasis (PFIC) are now well understood [39]. Animal models, particularly mouse models, have been extremely instructive in elucidating the functions of human ABC transporters but there is often a marked inconsistency between human and mouse pathologies, for example in CF and X-linked adrenoleukodystrophy [30,40]. In the case of cholestatic disease, this has proved informative, since transporter function could be studied in *abcb11* knockout mice without the development of secondary pathology but this disparity also emphasizes the importance and utility of studying the biochemistry of recombinant human ABCs [39].

In addition to roles associated with endogenous substrates, several ABC transporters are known to show relatively limited substrate specificity. This multi-drug transport capability is well established for ABCB1, ABCC1 and ABCG2 (and new drugs must be tested as transport substrates of these three promiscuous pumps) [41]. Two clinical correlates of multi-drug transport were explored: the first was the role ABC transporters play in drug metabolism. Andrew Owen [42,43] described mathematical approaches in pharmacokinetics (PK), contrasting population PK (starting with clinical data and fitting a model) with physiological PK (predicting clinical outcome from models of transport data). Such PK models can already be used to simulate different treatment regimens and predict how they might work for patients with different transporter SNPs (single-nt polymorphisms) [42,43]. Secondly, multi-drug transporters are partially responsible for cancer drug resistance, which causes tens of thousands of cancer deaths each year. The possibility of specifically targeting cancer stem cells expressing multi-drug pumps (e.g. ABCB1) was described by Beth Coyle [44]. By repurposing a hypertension drug, Vardenafil, inhibition of ABCB1-mediated drug resistance was achieved in cell lines established from difficult to treat children's brain tumours. Intriguingly, Vardenafil may not only overcome one particular challenge to brain tumour chemotherapy since it crosses the blood–brain barrier, but its inhibition of ABCB1 may also have a collateral benefit in reducing dissemination and invasion of drug resistant tumour cells [44]. Although ABCB1, C1 and G2 are the three usual suspects in multi-drug resistance, other ABC transporters also play roles in drug metabolism and beyond in cancer biology [41]. In this context, Viktor Hlaváč [45] presented emerging evidence for differential regulation of sub-class D ABC transporters associated with both positive and negative outcomes in diverse cancers.

## Structure and mechanism: an ideal *pas de deux*?

Visualizing the structure of ABC transporters at atomic resolution has become something of a holy grail in the field but an understanding of their dynamics is equally important [46]. Ironically, the first ABC-related structure of a soluble SBP was published 10 years before the ABC transporter family was defined [47]. Although many crystal structures have followed and SBPs are the best understood components of ABC transporters, they still hold a few surprises. Gavin Thomas [48] elegantly elucidated the molecular basis of substrate specificity in previously uncharacterized *E. coli* SBPs. Liganded crystals not only identified unexpected substrates but also revealed how the specificity can be changed dramatically by a single alteration in a residue distant to the binding site. Thorben Cordes followed with a fascinating presentation of mechanistic detail derived from single-molecule FRET studies of SBPs, challenging the classical ‘Venus Flytrap’ model [49,50].

Although important 3D insights have been obtained from EM and single particle analysis of full-length membrane transporters [51,52], high-resolution ABC crystal structures have been eagerly awaited. Membrane proteins are notoriously challenging for structural studies, the first task being to produce pure, preferably active protein; an appreciation of the magnitude of this undertaking is evident when considering the huge worldwide effort that has gone into characterizing P-gp [12] and the difficulties in heterologous expression of many plant ABC transporters [24]. In this context, Naomi Pollock [31] outlined different approaches to preparing recombinant CFTR for structural studies, emphasizing the importance of testing orthologues from different species and optimization of detergent solubilization [31]. Perhaps unsurprisingly, most structures obtained to date are of prokaryotic ABC transporters which are generally easier to express and purify in quantities amenable to crystallization, reviewed here by Kostas Beis [53], who presented a synthesis of structural and biophysical data for the anti-bacterial peptide transporter, McjD at the meeting.

Liz Carpenter [54] highlighted a different problem frequently faced by structural biologists that the proteins which crystallize most readily are not necessarily those for which functional information is available. ABCB10 was the first human ABC transporter to produce diffracting crystals in a structural genomics project but its precise function remains frustratingly obscure [25,54]. Although high resolution crystal structures of orphan transporters are helpful for generating homology models and can give insights into different conformational states, their utility is greatly enhanced when the substrates are known and where complementary biochemical or biophysical data are available.

An armoury of complementary approaches is now available to probe ABC transporter structure and mechanism and these can be particularly powerful when combined. For example, van Veen discussed the use of cysteine-reactive fluorescent reagents to investigate the catalytic cycle of MsbA in the framework of crystal structures [55] and Fraser Macmillan [46,56] used P-gp to explain how EPR spectroscopy can be employed in combination with site-directed spin labelling to measure distances and probe accessibility in ABC transporters. Where structures are available, MD simulations, together with homology modelling, offer the possibility to describe conformational dynamics, albeit over short time scales. Laura Domicevica and Philip Biggin [57] presented an analysis of P-gp drug binding using homology modelling with MD and Thomas Stockner [46] discuss here how EPR and MD can be combined to investigate the conformational flexibility of ABC transporters, comparing dynamic data for MsbA and P-gp with mechanistic predictions from crystal structures in different conformations. A promising complementary technique was introduced by Bavro [58]; X-ray radiolytic footprinting combined MS (XF–MS) though not yet applied to ABC transporters, offers valuable insights into the dynamics of membrane proteins; in particular XF–MS permits identification of structural waters and conformational changes in proteins and is applicable to protein complexes, which will interest many members of the ABC transport community.

## Conclusions and perspectives: whither ABCs?

In 40 years, ABC transporter research has evolved into a diverse, dynamic field with activity focused on both basic and translational issues. The meeting brought home how these extremes can inform and complement each other and how biochemistry and genetics are still very much at the heart of ABC transporter research. Much remains still to be discovered, encapsulated by two major questions:

1) What do all those transporters do? In medicine, GWAS (genome-wide association studies) and exome sequencing are likely to reveal ABC involvement in even more medical conditions, particularly rare diseases, whereas in yeast, bacteria and plants, forward genetic and chemical screens are predicted to uncover new functions for ABC transporters. Identifying substrates for these ‘orphan’ transporters will remain a major challenge as it has been for the g-protein coupled receptor community.

2) How do they all work? Emerging techniques have much to offer the field, including new methods for purification and reconstitution [59], single molecule and single liposome approaches [50,60,61], oxidative footprinting [58] and subnanometer cryoelectron microscopy [62]. Although we remain tantalized by the prospect of a series of high-resolution liganded stuctures in states comprising the entire catalytic cycle, with accompanying biophysical data, we should remain sanguine and appreciate that a single unifying mechanistic model of transport may not apply to this diverse family of intriguing proteins.

## Abbreviations

ABC: ATP-binding cassette
CF: cystic fibrosis
CFTR: cystic fibrosis transmembrane conductance regulator
GWAS: genome-wide association study
NBD: nt-binding domain
P-gp: permeability glycoprotein
PFIC: progressive familial intrahepatic cholestasis
PK: pharmacokinetics
SBP: substrate-binding protein
SUR: sulfonylurea receptor
TMD: transmembrane domain
XF–MS: X-ray radiolytic footprinting with mass spectroscopy
